# The role of robotic-assisted surgery in the management of rectal cancer: a systematic review and meta-analysis

**DOI:** 10.1097/JS9.0000000000001380

**Published:** 2024-03-27

**Authors:** Chenxiong Zhang, Hao Tan, Han Xu, Jiaming Ding

**Affiliations:** a Department of Anorectal Surgery, Yubei Hospital of Traditional Chinese Medicine, Chongqing Yubei District, Chongqing, People’s Republic of China; b Guangzhou University of Chinese Medicine, Guangzhou, People’s Republic of China

**Keywords:** health promotion, non-communicable diseases, rectal cancer, risk factors, robotic-assisted surgery

## Abstract

**Background:**

Rectal cancer poses a significant global health burden. There is a lack of concrete evidence concerning the benefits of robotic-assisted surgery (RAS) for rectal cancer surgery as compared to laparoscopic and open techniques. To address this gap, we conducted a meta-analysis to assess the intraoperative, postoperative, and safety outcomes of robotic surgery in this context.

**Research methodology:**

A search of MEDLINE, Scopus and the Cochrane Library. Randomized and non-randomized studies up to February 2, 2024 comparing robotic surgery versus laparoscopic or open surgery for rectal cancer. The outcomes of interest were operative time, blood loss, harvested lymph nodes, conversion rate, postoperative hospital stay, survival to hospital discharge, urinary retention rate, and anastomotic leakage rate. A random-effects meta-analysis was performed to pool means and dichotomous data to derive weighted mean differences and odds ratios, respectively.

**Results:**

A total of 56 studies were shortlisted after the study selection process with a total of 25 458 rectal cancer patients. From the intraoperative outcomes, RAS was significantly associated with an increased operative time (WMD: 41.04, *P*<0.00001), decreased blood loss (WMD: −24.56, *P*<0.00001), decreased conversion rates (OR: 0.39, *P*<0.00001), lesser stay at the hospital (WMD: −1.93, *P*<0.00001), and no difference was found in lymph nodes harvested. Similarly, RAS group had a significantly greater survival to hospital discharge (OR: 1.90, *P*=0.04), decreased urinary retention rate (OR: 0.59, *P*=0.002), and no difference was seen in anastomotic leakage rate.

**Conclusion:**

RAS demonstrates favorable outcomes for rectal cancer patients, contributing to global prevention and control efforts, health promotion, and addressing non-communicable disease risk factors. Further research and public awareness are needed to optimize RAS utilization in this context.

## Introduction

Highlights

- Our study is a systematic analysis of recent studies, and comprehensive evaluation concluded that robotic-assisted surgery (RAS) demonstrates favorable outcomes for rectal cancer patients, contributing to global prevention and control efforts, health promotion, and addressing non-communicable disease risk factors.
- This is the first systematic evaluation to validate the safety and feasibility of (RAS) and its application.
- RAS is defined as robotic-assisted surgery.

Colorectal cancer treatment primarily relies on surgical resection as it offers the most effective means for achieving curative resection, accurate staging, favorable prognosis, and other therapeutic considerations^1,2^. The primary goal of cancer surgery revolves around improving survival rates and quality of life for patients while minimizing potential adverse effects^3^. To enhance outcomes related to functional, oncological, surgical, patient-reported, and financial aspects of colorectal cancer treatment, it is crucial to advance surgical procedures^4^.

Over the past two decades, there has been a notable inclination toward the adoption of minimally invasive procedures^5^. Despite initial skepticism, this approach has gained significant momentum since its initial description in 1991 and has now become the established standard of care in Western countries for both benign and malignant colorectal diseases^5^. Numerous randomized trials have unequivocally demonstrated the superiority of laparoscopic surgery over open surgery for colon cancer treatment^6,7^. This preference stems from its associated benefits, such as reduced blood loss, faster recovery of bowel motility, and shorter hospital stays, all without compromising oncologic outcomes. As a result, laparoscopic surgery has become the established standard method for colon cancer treatment. Nevertheless, the laparoscopic approach is not without its inherent technical limitations such as a restricted range of motion for elongated instruments within the confined pelvic cavity, two-dimensional visual perception, diminished tactile sensitivity, and poor ergonomics.

To surmount these challenges, the use of robotic-assisted surgery (RAS) was undertaken with the aim of addressing these limitations. The da Vinci system (Intuitive Surgical, Sunnyvale, CA, USA) is the first system approved by the FDA in 2000^8^. Since the first robotic colectomy was performed in 2001, the use of robotics has been increasing ever since for both benign and malignant conditions. Because of the improved dexterity and the high-dimensional three-dimensional view, precise dissection and excellent exposure are observed with the use of robotic surgery for rectal cancers^9^.

The field of colorectal surgery has witnessed a remarkable transformation in the surgical management of rectal carcinomas with the advent of robotic surgery, leveraging cutting-edge technologies. This technique has gained significant popularity due to its potential advantages. However, despite the growing utilization of robotic surgery, the precise role it plays in colorectal cancer surgery remains largely uncertain. The question of whether robotic surgery offers substantial clinical benefits over laparoscopic surgery for the treatment of colorectal cancer remains unanswered. A recent meta-analysis comparing robotic and laparoscopic approaches for rectal cancers found no significant difference in overall survival and crucial postoperative complications between both groups^10^. Hence, we performed this systematic review and meta-analysis to compare RAS with conventional laparoscopic and open approaches to rectal cancers.

## Methods

This study was registered on the PROSPERO website (CRD42023430839). This systematic review and meta-analysis were performed in accordance with the Preferred Reporting Items for Systematic Reviews and Meta-analysis (PRISMA)^11^, Cochrane Collaboration guidelines^12^, and was in compliance with A MeaSurement Tool to Assess systematic Reviews (AMSTAR-2) Checklist^13^.

### Literature search and study selection

Databases such as MEDLINE, Scopus, and the Cochrane Library were searched from inception till 2nd February 2024. The following keywords were used in the search string: “rectal neoplasms”, “rectal cancers”, “robotic assisted”, “laparoscopic”, and “surgery”. The detailed search string is shown in Supplementary Material Table S1 (Supplemental Digital Content 1, http://links.lww.com/JS9/C308). Additionally, conference proceedings, www.clinicaltrials.gov, and bibliometrics of published articles were browsed to ensure no articles were missed.

Articles from the literature search were exported to Endnote Reference Library (Version X7.5; Clarivate Analytics, Philadelphia, Pennsylvania) software, where the duplicates were identified and removed. The remaining articles were then thoroughly reviewed by independent reviewers, ensuring that the selected articles met the defined eligibility criteria. The following inclusion criteria were used to shortlist studies: (1) adult patients with diagnosed rectal cancer, (2) robotic-assisted rectal cancer surgery being done, (3) compared with conventional laparoscopic or open surgery, and (4) randomized controlled trials and non-randomized studies. Studies published in the English language were shortlisted, and studies with small sample sizes (*n*<10) were excluded.

### Data extraction and quality assessment

Data extraction was extracted and verified by two reviewers. Any discrepancies were resolved through discussion and consensus. Further random ball sampling was done to include all the relevant studies. Data extracted from each study included study design, study population, sample size, number of patients in each group, general patient characteristics (age and gender), pathological characteristics, and primary and secondary endpoints. Following were the outcomes of interest: survival to hospital discharge, lymph nodes harvested, operative time, postoperative hospital stay, blood loss, conversion rate, urinary retention rate, and anastomotic leakage rate.

The quality assessment of RCTs was done using the risk of bias-2 tool (RoB-2) of Cochrane Collaboration^14^. ROBINS-I was utilized for the risk of bias evaluation of non-randomized studies^15^.

### Statistical analysis

Review Manager (version 5.4.1; The Nordic Cochrane Centre, The Cochrane Collaboration, 2020, Copenhagen) was used for all relevant meta-analyses of this study. A random-effects model was used due to the large number of studies, variability in study populations, differences in study designs and methods, and unknown sources of heterogeneity. This meta-analysis was performed to derive weighted mean differences (WMDs) and odds ratios (ORs) for continuous and dichotomous data, respectively. Publication bias was assessed by checking asymmetry in the funnel plots for outcomes with greater than 10 studies. A cumulative meta-analysis for the primary outcome of survival to hospital discharge was carried out using Open Meta-Analyst [Computer Program]. Egger’s regression test was performed to confirm the risk of publication bias. Subgroup analysis was performed based on a comparator (either laparoscopic or open) if the outcome had two or more studies of a specific subgroup. A *P*-value <0.05 was considered statistically significant for all outcomes. Heterogeneity was assessed with Higgin’s *I* ^2^ test. A value of *I* ^2^=25–50% was considered mild, 50–75% as moderate, and >75% as significant heterogeneity. Sensitivity analysis was performed by excluding the study comparing robotic approach with open approach, for outcomes with only one such study.

## Results

**Figure 1:**
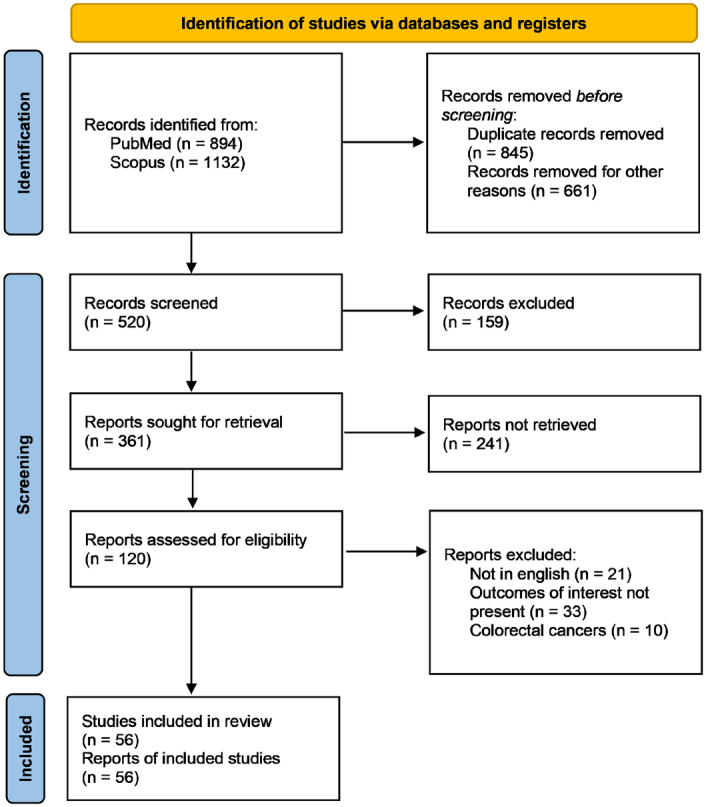
PRISMA flowchart.

A total of 2167 articles were identified from the literature search (Fig. 1). After deduplication and removing reviews, abstracts, editorials, and case reports, 126 studies were given a full-text evaluation, and 56 were finally included in the meta-analysis^16–70^. The risk of bias was low for 44 studies and the rest were moderate (Supplementary Fig. S1, Supplemental Digital Content 1, http://links.lww.com/JS9/C308 and Supplementary Fig. S2, Supplemental Digital Content 1, http://links.lww.com/JS9/C308).

### Patient characteristics

A total of 25 458 rectal cancer patients were present in the 56 included studies (11 218 in the robotic group, 13 848 in the laparoscopic, and 392 in the open). The mean age of patients in the robotic and control groups was 61.5±16.7 and 63.6±12.3 years, respectively. The male sex percentage was 65.8% of the robotic arm compared to 62.3% in the control (Table 1). American Society of Anaesthesiologists (ASA) Physical Status Classification ≥III grade was present in 14.4% of patients in the RG group and 20.1% in the control group. Neoadjuvant chemotherapy was increasingly used more in the robotic group as compared to the control (41.2% vs. 32.8%). The proportion of rectal cancer being lower was 40.2% in the robotic group and 39% in the control group. Detailed characteristics are present in Table 2.

**Table 1:** Patient characteristics of the included studies.

		Robotic-assisted group			Laparoscopic group			Open group		
Study	Study design	No.	Age	Sex, male	No.	Age	Sex, male	No.	Age	Sex, male
Ahmed *et al*., 2017^16^	PCD	99	69±2	71.7%	85	68±2	68.2%			
Ali *et al*., 2022^65^	RS	64	61.7±12	62.5%	91	62.7±11.2	64%			
Aselmann *et al*., 2018^17^	R-PCD	44	61.1±11.5	59.1%	41	65.1±12.0	58.5%			
Asklid *et al*., 2018^18^	RCS	72	65.4±10.4	59.7%	47	70.1±12.0	44.7%			
Baek *et al*., 2010^19^	PCD	41	65.6±11.3	61.0%	41	64.4±13.3	61.0%			
Baek *et al*., 2012^20^	PCD	154	59.1±12.2	68.2%	150	62.3±10.9	72.7%			
Baek *et al*., 2013^21^	RS	47	50.8±12.9	66.0%	37	61.8±12.8	75.7%			
Baik *et al*., 2008^22^	RCT	18	57.3±6.3	77.8%	18	62.0±9.0	77.8%			
Barnajian *et al*., 2014^23^	RS	20	62.5±11	60.0%	20	61.3±13	60.0%			
Bedirli *et al*., 2015^24^	RS	35	64.7±8.5	68.6%	28	60.4±7.1	67.9%			
Bedrikovetski *et al*., 2020^25^	RS	117	61±9.3	63.2%	1269	62.5±13.7	57.9%			
Bianchi *et al*., 2010^26^	PCD	25	63.5±9.3	72.0%	25	62.5±13.7	68.0%			
Bilgin *et al*., 2020^27^	R-PCD	72	59.0±11.1	58.3%	44	57.2±13.3	75.0%			
Chen *et al*., 2017^28^	RS	4744	NS	NS	5578	NS	NS			
Cho *et al*., 2015^29^	PCD	278	57.4±11.6	65.5%	278	58.3±10.4	66.2%			
Corbellini *et al*., 2016^70^	RCT	65	60.3±29.7	53.8%	40	60±33.5	57.5%	55	60.6±30.5	65.5%
Corrigan *et al*., 2018^30^	RCT	237	NS	67.9%	234	NS	67.9%			
Crolla *et al*., 2018^31^	RS	168	67.0±9.64	67.3%	184	68.1±10.7	56.0%			
D’Annibale *et al*., 2013^32^	RS	50	66.0±12.1	60.0%	50	65.7±11.6	60.0%			
de Jesus *et al*., 2016^33^	PCD	59	56.8±14.7	61.0%	41	55.5±16.7	58.0%			
de’Angelis *et al*., 2020^34^	PCD	50	64.4±14.7	66.0%	81	55.5±16.7	60.5%			
Esen *et al*., 2018^35^	PCD	100	59±11	60.0%	78	56±13	65.0%			
Feng *et al*., 2021^62^	RS	137	58.3±11.2	54.7%	137	59.5±11.2	59.1%			
Feng *et al*., 2022^66^	RCT	586			585					
Feng *et al*., 2022^68^	RCT	174	58.2 (9.6)	62.1%	173	59.5 (10.9)	65.3%			
Fernandez *et al*., 2013^57^	RS	13	67.9±2.1	100%	59	64.9±1.2	97%			
Feroci *et al*., 2016^36^	RS	53	64.5±12.1	50.9%	58	61.3±13.6	72.4%			
Garfinkle *et al*., 2019^37^	RS	154	61.9±13.5	68.8%	213	63.8±13.3	59.6%			
Ishihara *et al*. 2018^38^	PCD	130	61.3	58.0%	234	64.1	65.0%			
Jayne *et al*., 2017^39^	RCT	237	64.4±11.0	67.9%	234	65.5±11.9	67.9%			
Kang *et al*., 2013^40^	PCD	165	61.2±11.4	63.0%	165	60.4±11.8	58.8%			
Kethman *et al*. 2020^41^	Cohort	192	61.7	69.0%	206	62	63.4%			
Kim *et al*., 2012^42^	PCD	30	54.13±8.52	60.0%	39	56.85±11.14	51.3%			
Kim *et al*. 2016^43^	PCD	33	57.0±9.6	69.7%	66	58.2±9.8	69.7%			
Kim *et al*., 2018^44^	RCT	66	60.4±9.7	77.3%	73	59.7±11.7	71.2%			
Law*et al*., 2017^71^	PCD	220	63.5±9.3	67.3%	171	63.3±12.2	56.7%			
Lim *et al*., 2016^46^	RS	74	65.1±12.4	67.6%	64	65.8±11.1	71.9%			
Liu *et al*., 2019^45^	RS	80	62±9.64	66.3%	116	59.57±10.3	62.1%			
Megevand *et al*., 2019^60^	PCD	35	70	65.7%	35	66	51.4%			
Ose *et al*., 2021^64^	RS	713	67.3±10.1	66.3%	1163	67.6±10.3	61.4%	205	67±10.2	64.4%
Park *et al*., 2011^47^	PCD	52	57.3±12.3	53.8%	123	65.1±10.3	56.9%			
Park *et al*., 2012^47^	RS	40	57.3±12.1	70%	40	63.3±10.6	62.5%			
Park *et al*., 2021^67^	RS	118	60.0±10.8	76.3%	118	60.3±11.1	73.7%			
Park *et al*., 2023^69^	RCT	151	65.5±11.4	64.2%	144	67.2±10.1	68.8%			
Patriti *et al*., 2009^48^	PCD	29	68±10	57.7%	37	69±10	33.3%			
Ramji *et al*., 2016^49^	RS	26	62.1±9.1	73%	27	63.7±11.2	70.0%			
Rouanet *et al*., 2018^50^	RS	200	59.5±10	65.5%	200	62±8.5	68.0%			
Shiomi *et al*., 2016^52^	RS	127	62±9.3	73.2%	109	654±10	59.6%			
Silva-Velazco *et al*., 2017^51^	RS	66	56±13.8	75.8%	118	59.8±9.8	55.9%			
Somashekhar *et al*., 2015^58^	PCD	25	56.36 ± 8.21	68%				25	59.56 ± 5.75	60%
Song *et al*., 2021^61^	RS	70	59.2 ± 37.8	65.7%	29	58.7± 34.1	82.6%			
Sugoor *et al*., 2018^53^	PCD	100	48.7±15.3	76.0%	113	49.2±14.6	61.1%			
Tilney *et al*., 2019^63^	PCD	204	64.4±11.4	77%	133	66.6±12.2	62.4%			
Valverde *et al*., 2017^54^	PCD	65	67±11	65.0%	65	65±10	69.0%			
Yamaguchi *et al*., 2016^55^	RS	203	64.8±10.8	69.0%	239	65.9±10.8	64.4%			
Yang *et al*., 2018^59^	RS	91	60±13.5	48.4%	102	59.1±11.6	59.8%	107	62.2±11.4	57.9%

PCS, prospectively collected data; R-PCD, a retrospective analysis of prospectively collected data; RCT, randomized controlled trial; RS, retrospective study.

**Table 2:** Baseline characteristics of the included studies.

		Events/participants				
Outcome	Number of studies	Robotic	Laparoscopic	Number of studies	Robotic	Open
Sex, male	52	3793/5733 (66.2%)	4789/7662 (62.5%)	4	569/894 (63.6%)	247/392 (63%)
American Society of Anesthesiologists (ASA) score						
1 class	39	1250/4142 (30.1%)	1636/5790 (28.2%)	2	240/713 (33.7%)	45/260 (17.3%)
2 class	39	2197/4142 (53%)	3014/5790 (52.1%)	2	424/713 (59.5%)	141/260 (54.3%)
3 class	39	576/4142 (13.9%)	1057/5790 (18.3%)	2	109/713 (15.3%)	72/260 (27.7%)
4 class	31	19/3392 (0.6%)	52/5124 (1.0%)	1	1/713 (0.1%)	0/205 (0%)
Neoadjuvant therapy	29	1493/3626 (41.2%)	1806/5472 (33%)	2	163/804 (20.3%)	90/312 (28.8%)
Tumor location						
Upper rectum	14	300/1733 (17.3%)	607/3201 (19%)	2	12/116 (10.1%)	17/132 (12.5%)
Middle	15	963/1948	1565/3416 (45.8%)	2	54/116 (49.1%)	68/132 (51.5%)
Lower	20	1026/2552 (40.2%)	1491/4032 (37%)	2	32/116 (27.6%)	28/132 (21.2%)

### Intraoperative and postoperative outcomes

**Figure 2:**
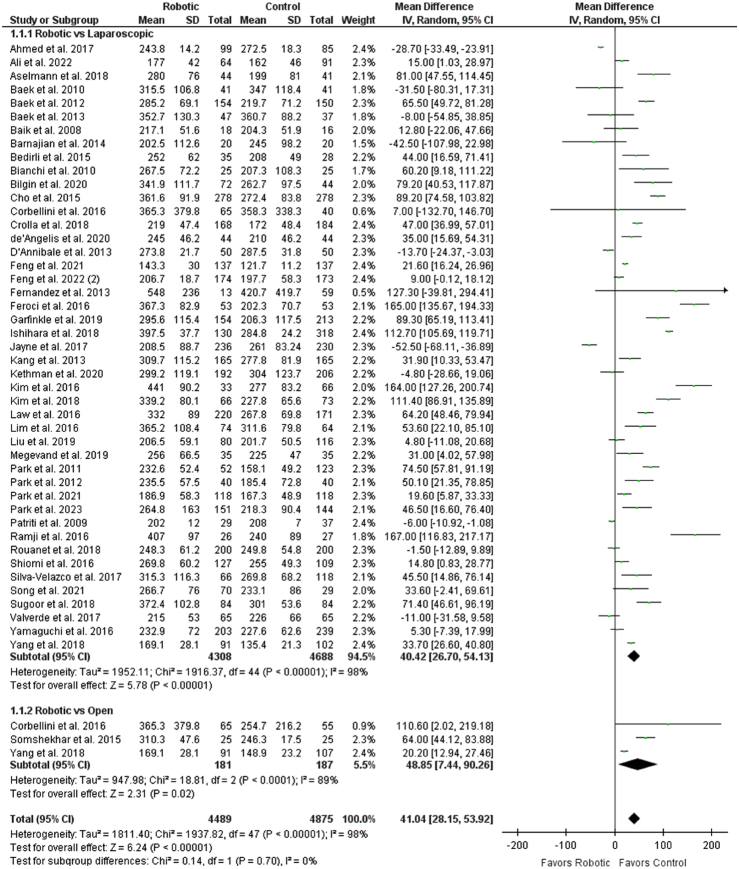
Forest plot for operative time.

**Figure 3:**
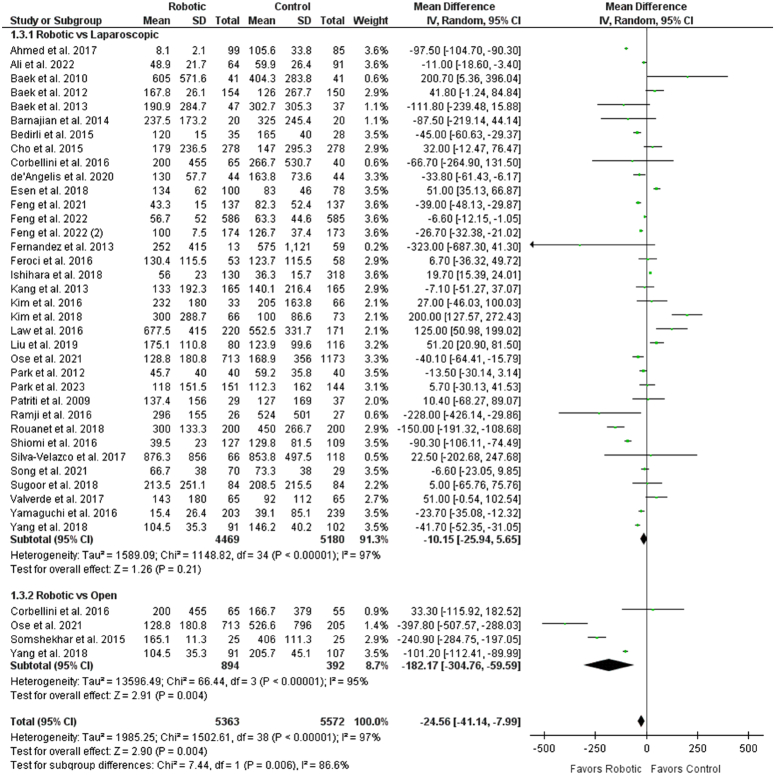
Forest plot for intraoperative blood loss.

**Figure 4:**
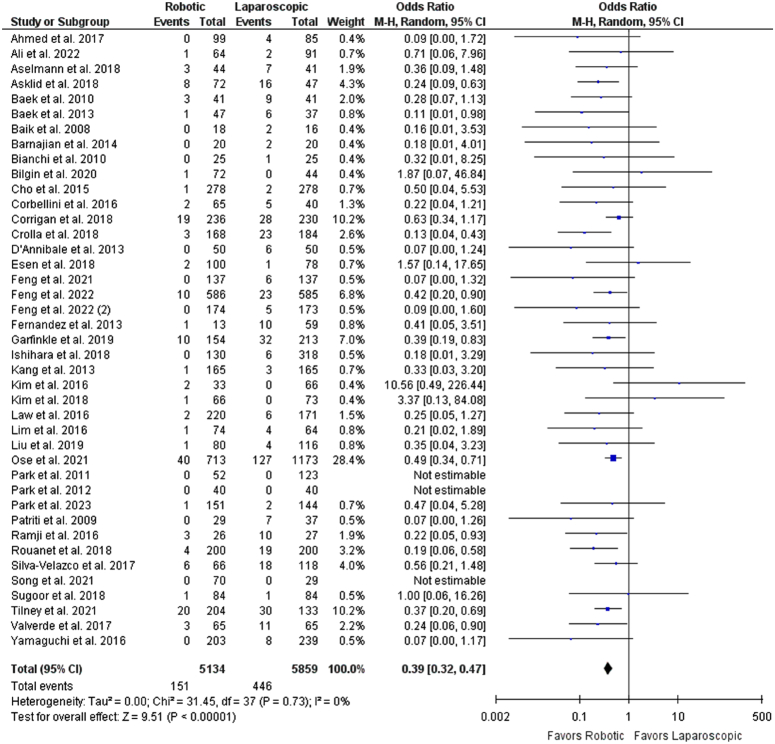
Forest plot for open surgery conversion rates.

**Figure 5:**
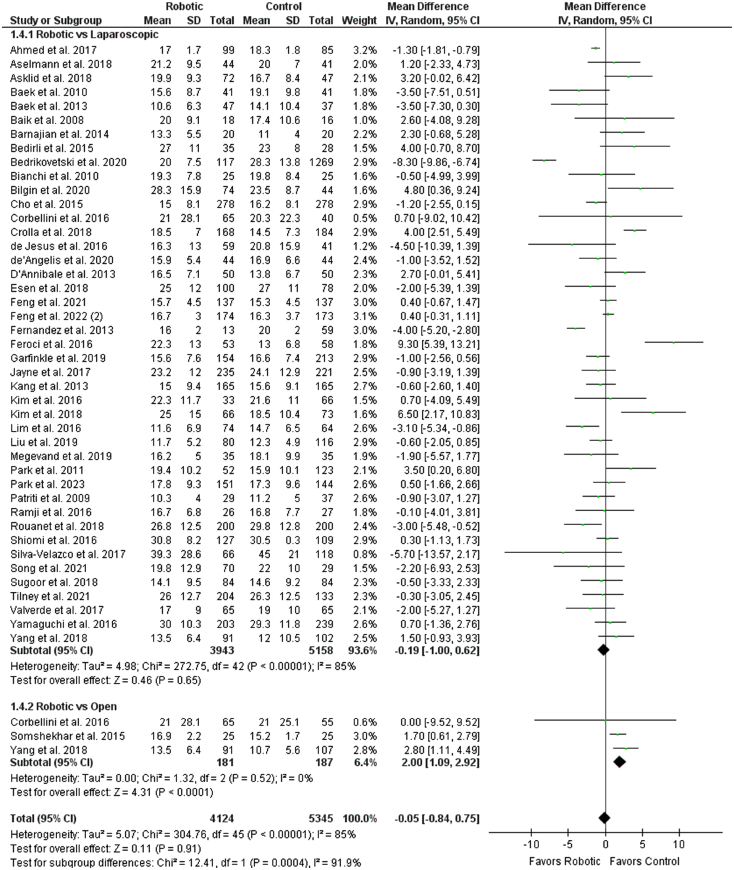
Forest plot for the number of harvested lymph nodes.

**Figure 6:**
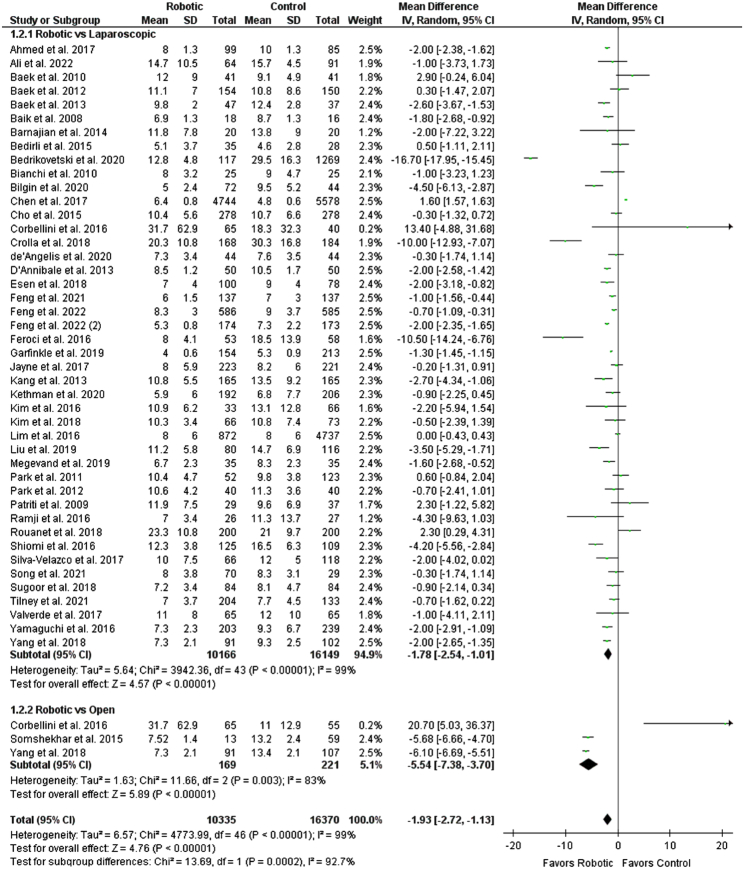
Forest plot for postoperative hospital stay.

Intraoperative outcomes consisted of operative time in minutes, intraoperative blood loss (ml), conversion to open surgery, and number of harvested lymph nodes. Robotic group was significantly associated with increased operative time when compared with the control group (WMD: 41.04 [28.15, 53.92], *P*<0.00001; *I* ^2^=98%) (Fig. 2). Upon subgroup analysis, robotic group had a greater operative time when compared with laparoscopic (WMD: 40.42 [26.70, 54.13], *P*<0.00001; *I* ^2^=98%) and open groups (WMD: 48.85 [7.44, 90.26], *P*=0.02; *I* ^2^=89%) independently. Operative time was compared between robotic and laparoscopic groups for randomized controlled trials. No significant association was seen across eight RCTs (WMD: 15.02 [−10.05, 40.08], *P*=0.24; *I* ^2^=97%) (Supplementary Fig. S3, Supplemental Digital Content 1, http://links.lww.com/JS9/C308). Patients in the robotic group had a significantly decreased loss of blood when compared with the control group (WMD: −24.56 [−41.44, −7.99], *P*<0.00001; *I* ^2^=97%) (Fig. 3). However, subgroup analysis showed no significant difference between robotic and the laparoscopic groups (WMD: −10.15 [−25.94, 5.65], *P*=0.21; *I* ^2^=97%) and decreased blood loss levels with robotic group when compared with open surgery (WMD: −182.17 [−304.76, −59.59], *P*=0.004; *I* ^2^=95%). Blood loss was also compared between robotic and laparoscopic groups for randomized controlled trials. No significant association was seen across six RCTs (WMD: 8.74 [−50.53, 68.01], *P*=0.77; *I* ^2^=99%) (Supplementary Fig. S4, Supplemental Digital Content 1, http://links.lww.com/JS9/C308). The robotic group was associated with decreased conversion rates to open surgery as compared to the laparoscopic approach (OR: 0.39 [0.32, 0.47], *P*<0.00001; *I* ^2^=0%) (Fig. 4). No significant difference was observed between the robotic and control groups comparing the number of harvested lymph nodes (WMD: −0.05 [−0.84, 0.75], *P*=0.91; *I* ^2^=85%) (Fig. 5). However, subgroup analysis showed robotic group to be significantly superior to open group in harvesting lymph nodes (WMD: 2.00 [1.09, 2.92], *P*<0.0001; *I* ^2^=0%). Postoperative hospital stay was found to be significantly lower in the robotic group as compared to the control group (WMD: −1.93 [−2.72, −1.13], *P*<0.00001; *I* ^2^=99%) (Fig. 6). Upon subgroup analysis, robotic approach was found to be superior to laparoscopic group (WMD: −1.78 [−2.54, −1.01], *P*<0.00001; *I* ^2^=99%) and open surgery group (WMD: −5.54 [−7.38, −3.70], *P*<0.00001; *I* ^2^=83%) as it had a lesser postoperative hospital stay.

### Safety outcomes

**Figure 7:**
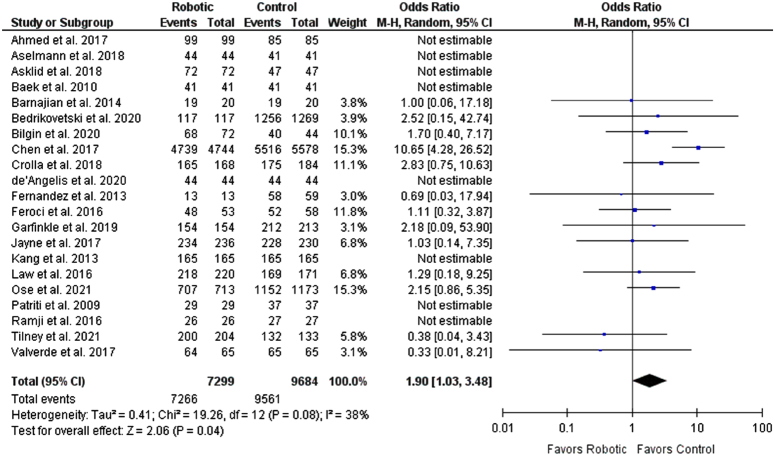
Forest plot for survival to hospital discharge.

**Figure 8:**
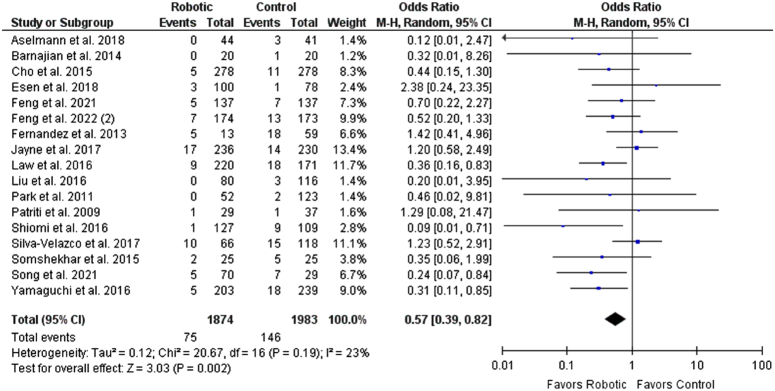
Forest plot for urinary retention rate.

**Figure 9:**
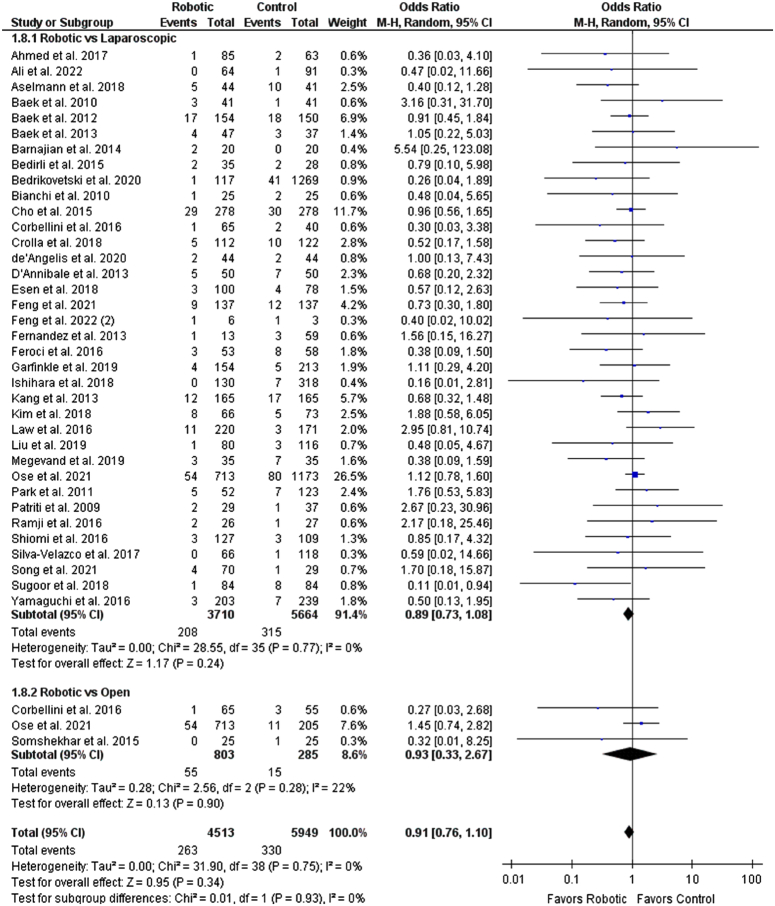
Forest plot for anastomotic leakage rate.

Survival to hospital discharge, urinary retention rate, and anastomotic leakage rate were included in the safety outcomes. The robotic group was significantly associated with increased survival at discharge from the hospital when compared with the control group (OR: 1.90 [1.03, 3.48], *P*=0.04; *I* ^2^=38%). For urinary retention rate, patients in the robotic group had significantly lesser urinary retention rates than the control group (OR: 0.59 [0.39, 0.82], *P*=0.002; *I* ^2^=23%). A sensitivity analysis was carried out removing Somashekhar *et al*.^58^ as it was the only study comparing the robotic group with open surgery (OR: 0.57 [0.39, 0.84], *P*=0.005; *I* ^2^=26%) (Supplementary Fig. S5, Supplemental Digital Content 1, http://links.lww.com/JS9/C308). No significant difference was observed for anastomotic leakage rate between the groups (OR: 0.91 [0.76, 1.10], *P*=0.34; *I* ^2^=0%). Additionally, no subgroup differences were observed for the laparoscopic and the open groups (*P*=0.93) (Figs 7–9).

### Publication bias and cumulative meta-analysis

For the assessment of publication bias, funnel plots were generated to ensure there was no asymmetry in the funnel plot. For the outcomes of operative time, blood loss, and postoperative hospital stay, asymmetry was observed in the funnel plots; thus, Egger’s regression test was performed to confirm the risk of publication bias. After Egger’s test, publication bias was confirmed for only postoperative hospital stay length (*P*=0.004). All funnel plots are shown in Supplementary Material Figures S6–S13 (Supplemental Digital Content 1, http://links.lww.com/JS9/C308). A umulative meta-analysis stratifying by year was performed to determine the outcome of survival to hospital discharge. This analysis showed the overall effect size to converge over time, indicating consistency of the pooled result (Supplementary Fig. S14, Supplemental Digital Content 1, http://links.lww.com/JS9/C308).

## Discussion

We performed this meta-analysis by pooling all the data to date on robotic surgery and by including the most recent randomized controlled trials on this topic. In this meta-analysis, many significant findings were established. The survival rate for hospital discharge was found to be significantly higher in the robotic group. The robotic group was associated with an increased operative time, decreased postoperative hospital stay, decreased intraoperative blood loss, decreased conversion rates, and a decreased urinary retention rate.

A study found that robotic techniques in surgery offer visual advantages, including binocular vision with three-dimensional imaging, increased freedom of movement, and an ergonomic position. Robotic surgery reduces tremors and human errors, improving maneuverability. The learning curve is like laparoscopic training, and it may be easier to learn than laparoscopic surgery. Robotic surgery enables minimally invasive approaches even in challenging procedures or anatomically difficult regions, benefiting less experienced surgeons^72,73^.

In many observational studies and meta-analyses, robotic surgery for rectal cancers significantly reduced open conversions, postoperative complications, hospital stays, and urinary function^74,75^. However, the ROLARR trial conducted by Jayne *et al.* ^39^ found no significant differences in these outcomes. It overestimated the open surgery conversion rate for both the robotic and the laparoscopic group. Hence, a more robust COREAN trial^40^ found the conversion rate to be 1.2% in the laparoscopic group. Our meta-analysis with over 10 000 patients for this outcome found the open surgery conversion rate to be significantly lower in the robotic group. These findings are consistent with a recent meta-analysis conducted by Safiejko *et al*.^10^. Robotic surgery could provide higher surgical quality and avoid these open conversions, consistent with the technical advantages of robotic surgery. Robotic systems offer three-dimensional high-definition visualization, providing surgeons with a detailed and magnified view of the surgical field. This enhanced visibility can be particularly advantageous in rectal surgeries where precise dissection is crucial. Improved visualization may reduce the likelihood of complications, leading to a lower conversion rate^39^. Additionally, robotic systems offer ergonomic advantages, allowing surgeons to operate from a console in a comfortable seated position. This can lead to reduced fatigue during lengthy procedures, potentially decreasing the likelihood of conversion due to surgeon exhaustion^29,40^.

The decreased intraoperative blood loss seen in this meta-analysis is similar to the findings of previous RCTs(22,48). Robotic surgery was associated with decreased hospital stay in our meta-analysis. Patients undergoing surgery for rectal cancers by the robotic approach recover faster with lesser postoperative complications, leading to longer hospital stays. These findings are consistent with previous studies^59,62,68^. Additionally, the minimally invasive nature of robotic surgery involves smaller incisions compared to open surgery. This results in less tissue trauma, reduced pain, and quicker recovery^76^.

In our meta-analysis, no difference was seen in the number of harvested lymph nodes. The mean number of retrieved lymph nodes is an important criterion for judging whether the tumor will be cured. In 1990, the World Congress of Gastroenterology in Sydney recommended the removal of 12 lymph nodes^77^. These findings are consistent with recent RCTs conducted by Park *et al*.^69^ and Feng *et al*.^68^. A previous meta-analysis also found no significant association between the harvested lymph nodes and the robotic approach^10^. However, a study conducted in 2022 found increased lymph nodes harvested with the robotic approach^65^. These findings might be different from our pooled analysis due to the retrospective nature of the study.

According to our findings, anastomotic leaking was not significantly different between the robotic and the laparoscopic surgery groups. The 3D dimension and articulating equipment used in robotic surgery may make anastomosis easier. However, pooled analysis failed to match this hypothesis. These findings were also reported in the meta-analysis conducted by Safiejko *et al*.^10^.

Our meta-analysis found a significant association between increased survival to hospital discharge and the robotic approach. These findings are novel, as previous meta-analyses have failed to show this association^10^. This might be because we have performed our meta-analysis with recent large-scale studies showing lower mortality with the robotic approach^63,64^.

The robotic approach emerges as the most favorable option for managing rectal cancer when compared to open, laparoscopic, or transanal techniques, as it delivers the finest blend of oncological, functional, and patient recovery outcomes. The digital interface of surgical robots enables a shift in the paradigm of surgical training, facilitating shorter learning curves that are more comprehensive and, notably, reducing the morbidity and mortality associated with them. It is imperative for surgical societies to take the initiative in this transformative process and establish efficacious training programs for colorectal robotic surgery.

Our study is not without its limitations. We conducted this meta-analysis by pooling observational studies and randomized controlled trials in which patients have been matched according to different variables. Few studies have kept in place a 1:1 randomization. Neoadjuvant chemotherapy was significantly greater in the robotic arm, which may lead to edema and fibrotic changes, thus influencing the outcomes. RAS for rectal cancers, in comparison to laparoscopic and open approaches, is available at a higher cost, thus influencing the lower availability of the procedure. The studies conducted for the open approach were mostly in languages other than English; hence, they were excluded from our meta-analysis. Laparoscopic is the conventional approach to rectal cancers nowadays hence, a smaller number of studies were found comparing robotic and open surgery.

## Conclusion

Robotic-assisted techniques showed numerous differences as compared to laparoscopic and open techniques, as it significantly increased the operative time. However, the robotic group showed decreased intraoperative blood loss, reduced hospital stays, significantly lower conversion of the procedure to open surgery, lower risk of urinary risk retention, and increased survival to discharge rate.

## Ethical approval

My research is a meta-analysis that integrates existing data and does not involve experiments on humans or animals, so it is not applicable, and there are no ethical issues.

## Consent

Not applicable.

## Sources of funding

Not applicable. This is a systematic review and meta-analysis that does not apply and does not exist to patients.

## Author contribution

C.Z., H.T., and H.X.: conceived the study and carried out the research; C.Z. and H.T.: prepared the first draft of the manuscript; C.Z., H.T., and J.D.: directed the manuscript to completed. All authors were involved in the revision of the draft manuscript and have agreed to the final content.

## Conflicts of interest disclosure

The authors declare that they have no conflicts of interest and no financial interests related to the material of this manuscript.

## Research registration unique identifying number (UIN)

CRD42023430839

## Guarantor

The scientific guarantor of this publication is Chenxiong Zhang from the Department of Anorectal Surgery, Yubei Hospital of Traditional Chinese Medicine/Guangzhou University of Chinese Medicine.

## Data availability statement

Data sharing is not applicable to this article as no new data were created or analyzed in this study.

## Provenance and peer review

Not commissioned, externally peer-reviewed.

## Supplementary Material

SUPPLEMENTARY MATERIAL
