# Supplementary material for: The role of robotic-assisted surgery in the management of rectal cancer: a systematic review and meta-analysis
Source: Int J Surg. 2024 Mar 27;110(10):6282–96. doi: 10.1097/JS9.0000000000001380 (PMC11487048; doi:10.1097/JS9.0000000000001380)

**Supplementary Material**

**Table S1. Detailed search strategy.**

| **PubMed (n=956)** | **("rectal cancer"[All Fields] OR "CRC"[All Fields] OR "rectal carcinoma"[All Fields]) AND ("robotic"[All Fields] OR "robot"[All Fields] OR "robotic-assisted"[All Fields]) AND ("laparoscopic"[All Fields] OR "laparo*"[All Fields])** |
| --- | --- |
| **Scopus (n=1211)** | **("rectal cancer" OR "CRC" OR "rectal carcinoma") AND ("robotic" OR "robot" OR "robotic-assisted") AND ("laparoscopic" OR laparo*)** |

**Figure S1. Quality assessment of randomized controlled trials.**

**Figure S2. Quality assessment of non-randomized studies.**

**Figure S3. Comparison of operative time for randomized controlled trials.**

**
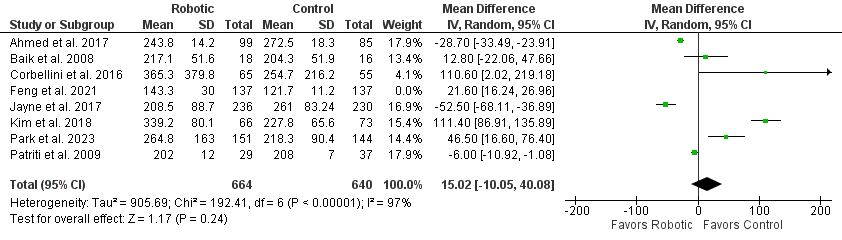
**

**Figure S4. Comparison of blood loss for randomized controlled trials.**

**
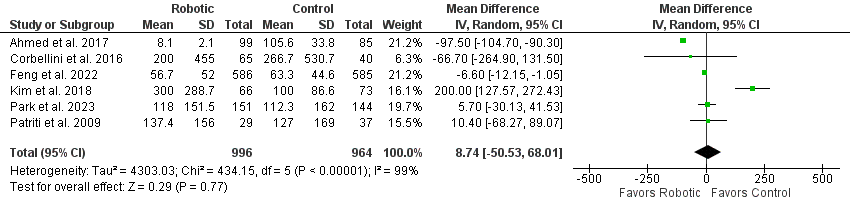
**

**Figure S5. Sensitivity analysis of urinary retention rate.**

**
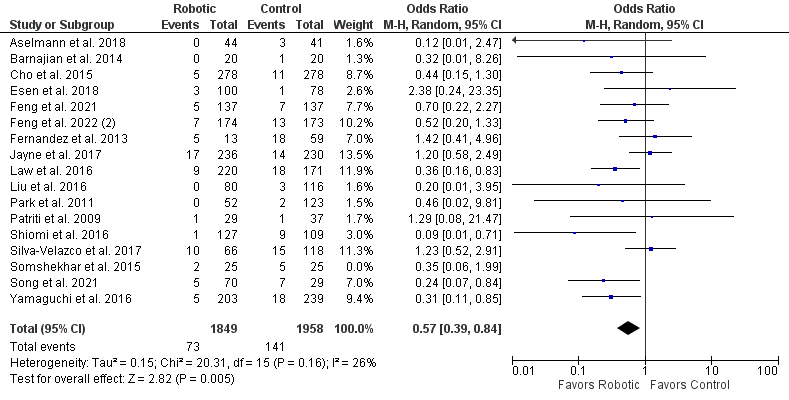
**

**
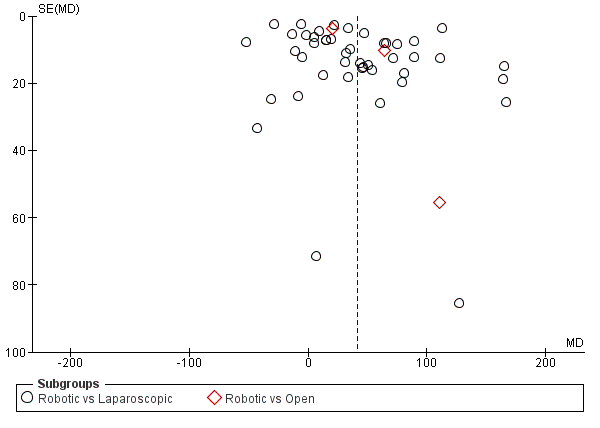
**

**Figure S6. Funnel plot of operative time.**

**
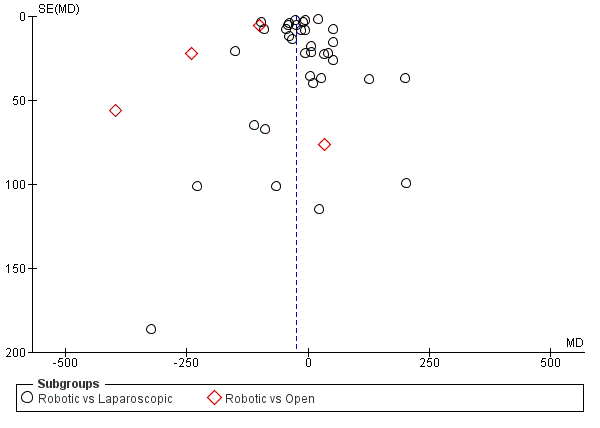
**

**Figure S7. Funnel plot of blood loss.**

**
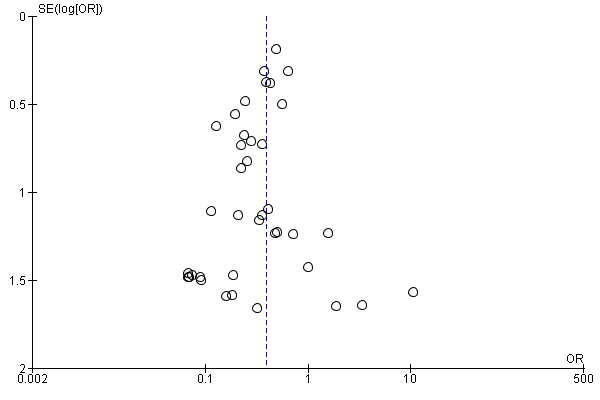
**

**Figure S8. Funnel plot of conversion rate.**

**
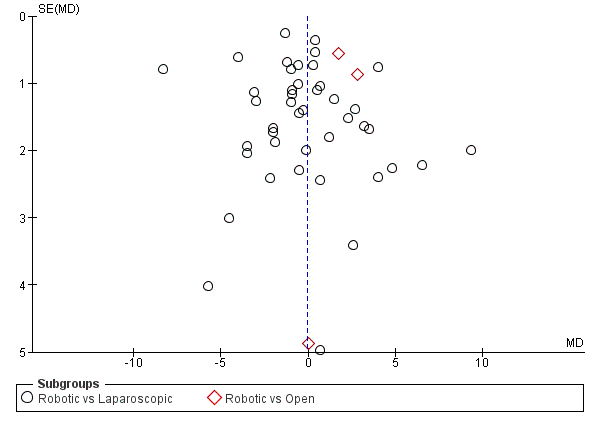
**

**Figure S9. Funnel plot of lymph nodes harvested.**

**
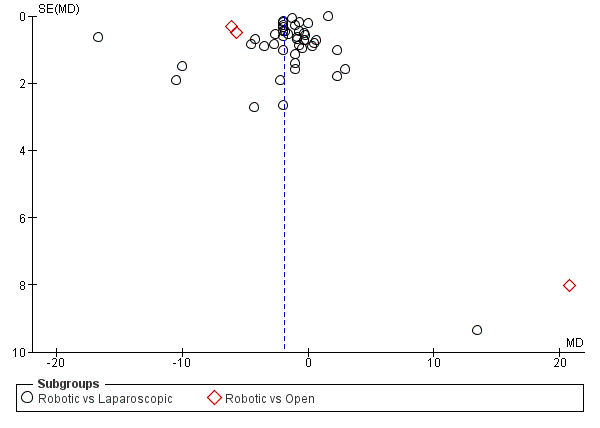
**

**Figure S10. Funnel plot of hospital stay.**

**
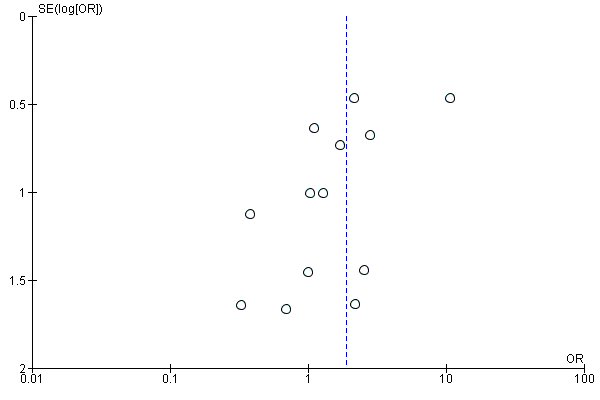
**

**Figure S11. Funnel plot of survival to hospital discharge.**

**
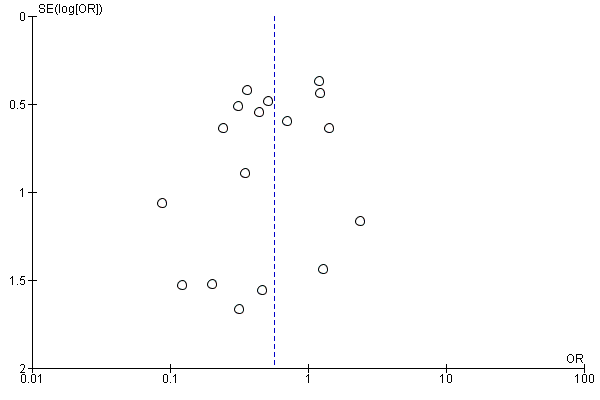
**

**Figure S12. Funnel plot of urinary retention rate.**

**
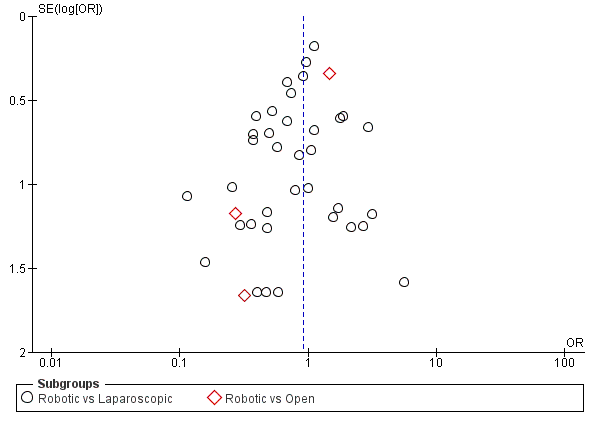
**

**Figure S13. Funnel plot of anastomotic leakage rate.**

**Figure S14. Funnel plot of cumulative meta-analysis of survival to hospital discharge.**


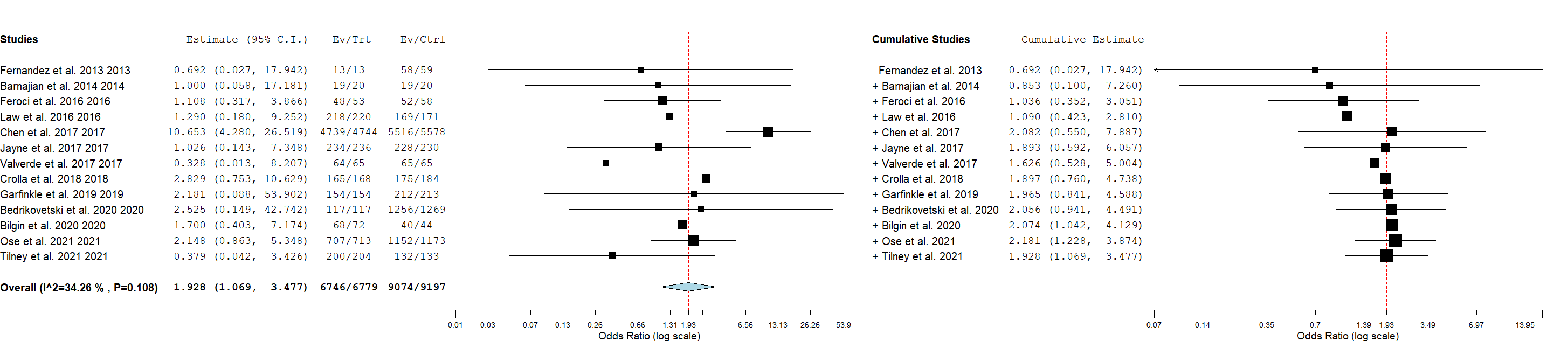

Supplement: SUPPLEMENTARY MATERIAL [file js9-110-6282-s001.docx]
